# Role of Exosomal miRNAs and the Tumor Microenvironment in Drug Resistance

**DOI:** 10.3390/cells9061450

**Published:** 2020-06-11

**Authors:** Patrick Santos, Fausto Almeida

**Affiliations:** Department of Biochemistry and Immunology, Ribeirão Preto Medical School, University of São Paulo, 3900 Bandeirantes Avenue, São Paulo, SP 14049-900, Brazil; patricksantos@usp.br

**Keywords:** exosomal miRNAs, chemoresistance, therapy resistance, extracellular vesicles

## Abstract

Tumor microenvironment (TME) is composed of different cellular populations, such as stromal, immune, endothelial, and cancer stem cells. TME represents a key factor for tumor heterogeneity maintenance, tumor progression, and drug resistance. The transport of molecules via extracellular vesicles emerged as a key messenger in intercellular communication in the TME. Exosomes are small double-layered lipid extracellular vesicles that can carry a variety of molecules, including proteins, lipids, and nucleic acids. Exosomal miRNA released by cancer cells can mediate phenotypical changes in the cells of TME to promote tumor growth and therapy resistance, for example, fibroblast- and macrophages-induced differentiation. Cancer stem cells can transfer and enhance drug resistance in neighboring sensitive cancer cells by releasing exosomal miRNAs that target antiapoptotic and immune-suppressive pathways. Exosomes induce drug resistance by carrying ABC transporters, which export chemotherapeutic agents out of the recipient cells, thereby reducing the drug concentration to suboptimal levels. Exosome biogenesis inhibitors represent a promising adjunct therapeutic approach in cancer therapy to avoid the acquisition of a resistant phenotype. In conclusion, exosomal miRNAs play a crucial role in the TME to confer drug resistance and survivability to tumor cells, and we also highlight the need for further investigations in this promising field.

## 1. Introduction

Primary human tumors consist of a mixture of genetically and phenotypically distinct cellular subpopulations. The initial conversion of a non-malignant cell to a malignant one is driven by genetic, epigenetic, and phenotypic changes that lead to cellular overgrowth, suppression of death signals, induction of angiogenesis, and resistance to therapy. Genomic instability, such as changes in chromosome number and structure, is one of the causes of tumor heterogeneity and is critical to cancer initiation and progression [1]. The genetic diversity of cells during cancer initiation may be the result of accumulated mutations from exogenous sources, such as UV radiation and/or endogenous processes—for example, a mistake during DNA replication [2]. Aside from the linear nature of cancer initiation followed by tumor progression, tumorigenesis is a dynamic process and continues to evolve to maintain tumor heterogeneity.

Beyond genetic diversity, the tumor microenvironment (TME) is crucial for tumor heterogeneity as it shapes cancer and non-cancer cell phenotypes [3]. The TME comprises different cell types, such as leukocytes, fibroblasts, and endothelial cells, within the tumor or in the tumor surroundings and is sustained by a vascular network and the extracellular matrix [4]. The interactions between tumor and non-tumor cells within the TME occur through direct cell-to-cell contact and secretion of soluble molecules, such as cytokines and chemokines [5]. Due to its heterogeneity and adaptability, the TME has been associated with the maintenance of the malignant behavior of cancer cells throughout different tumoral stages [6,7]. Additionally, the TME plays an important role in protecting cancer cells from drug therapy via crosstalk between cancer cells and surrounding cells.

Recently, the transport of biological mediators by extracellular vesicles (EVs) has received substantial attention and emerged as a key messenger in intercellular communication [8,9]. EVs are small, double-layered lipid vesicles released from the membranes of almost all cell types [10]. EVs carry lipids, proteins, and nucleic acids, such as DNA, mRNA, miRNA, and other non-coding RNAs, that can affect multiple processes in recipient cells, including gene expression changes and activation of several signaling pathways [11]. Three different classes of EVs have been identified based on their biogenesis. Ectosomes originate from the plasma membrane (100–1000 nm in diameter), apoptotic bodies are generated from cells undergoing apoptosis (50–2000 nm in diameter), and exosomes originate from endocytosis into multivesicular bodies (30–100 nm in diameter) [5,10,12].

**Figure 1:**
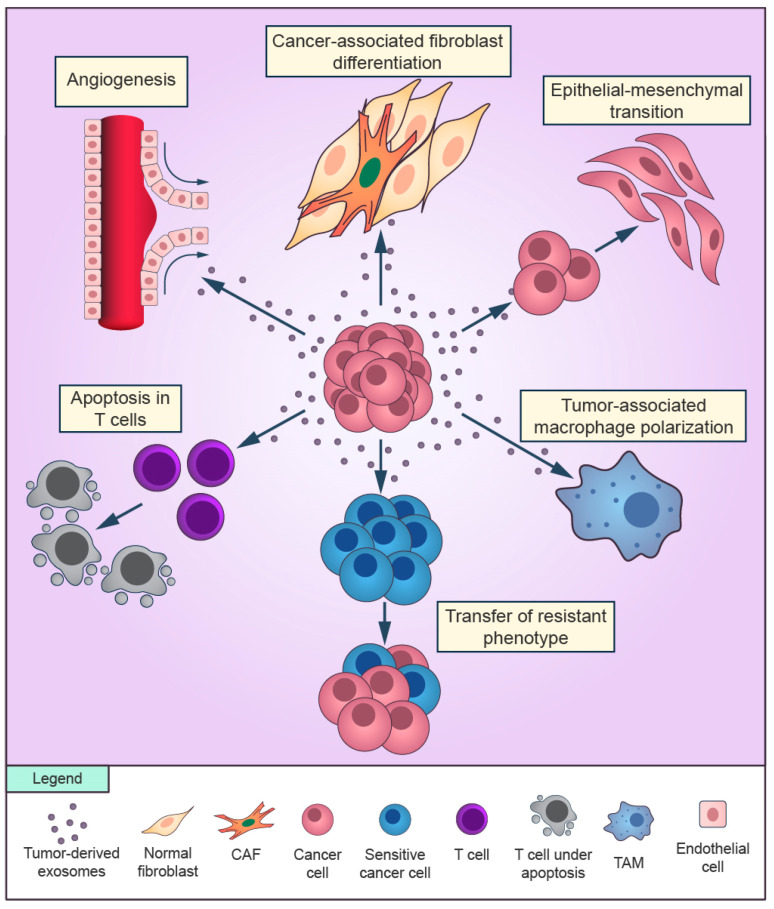
Overview of the role of cancer-derived exosomes in the tumor microenvironment. Exosomes secreted by cancer cells can promote cancer-associated differentiation in adjacent fibroblasts and macrophages polarization towards a tumor-supportive phenotype. Cancer cells can release exosomes that induce the epithelial-mesenchymal transition in other cancer cells and transfer the resistant phenotype to sensitive cells in the surroundings. Cancer-derived exosomes are able to promote angiogenesis and to suppress the antitumor immune response of T cells.

Currently, the aspects of tumor-derived exosomes in cancer progression and therapy resistance are the major focus of EV-associated pathology research [9]. Tumor-derived exosomes are involved in several hallmarks of cancer, including inflammatory responses, angiogenesis, evasion of apoptosis, cell proliferation, immune suppression, invasion, and metastasis [5,13,14] (Figure 1). Tumoral exosomes influence a plethora of cellular and molecular processes in the TME during acquired drug resistance in cancer cells, including the cell cycle, DNA repair, immune system surveillance, and the epithelial-mesenchymal transition (EMT) [15]. Drug resistance induced by tumoral exosomes is mediated through different routes, such as direct drug export, transport of drug efflux pumps, and signaling of miRNAs [16,17]. 

Exosomal miRNAs released by cancer cells can directly induce drug resistance in the tumor surroundings from a drug-resistant cell to a sensitive counterpart, or cancer exosomes can interact and deliver miRNAs to TME cells that will modulate a drug resistance response in the area [18]. The exosomal miRNA horizontal transfer of a resistant phenotype to sensitive cancer cells has been reported in different types of tumors with a wide variety of anticancer drugs [12,19]. However, how exosomal miRNAs interact with the TME to confer drug resistance properties to cancer cells is not fully understood. Here, we discuss the recent developments in mechanisms mediated by tumoral exosomes, focusing on miRNA interactions and signaling in the TME that confer survivability and a therapy-resistant phenotype upon tumor cells.

## 2. TME as a Mediator of Drug Resistance

### 2.1. Drug Resistance Overview

Despite the development of effective drugs for cancer treatment and their initial positive responses, drug resistance is still a critical limiting factor for the achievement of cures in cancer patients [20]. Drug resistance can be classified as intrinsic or acquired. Intrinsic drug resistance can be attributed to pre-existing resistant cancer cells before treatment and associated with drug breakdown, alterations in drug transport, and reduced interaction between the drug and its molecular target [21]. Acquired drug resistance constitutes a group of cells that emerge resistant from a population that was previously drug-sensitive, resulting in tumor regrowth [22]. Genetic, epigenetic, transcriptomic, and proteomic changes in cancer cells cause tumor heterogeneity, which is directly associated with acquired drug resistance [23]. Many tumors become resistant due to a combination of intrinsic and acquired drug resistance factors [20]. Moreover, a drug-sensitive tumor that contains at least one resistant cell clone can acquire drug resistance by clonal expansion [21]. 

### 2.2. Drug Resistance Mediated by Cancer-Associated Fibroblast Exosomal miRNAs

**Figure 2:**
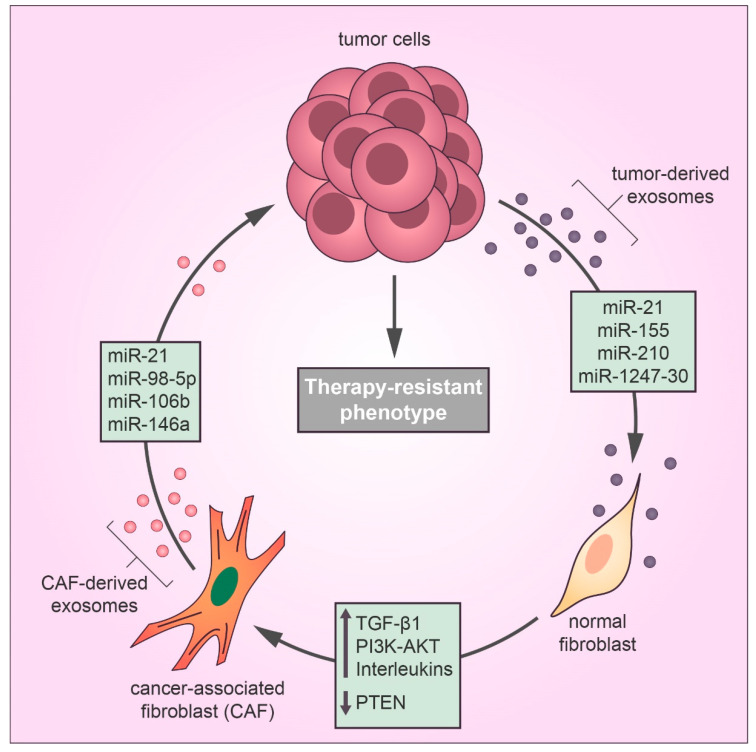
Exosomes secreted by cancer cells transfer miRNAs to fibroblasts in the tumor microenvironment that induce the cancer-associated fibroblast (CAF) differentiation, thereby exosomal miRNAs derived from CAFs confer drug resistance in cancer cells by inducing metastasis and proliferation and inhibiting the antitumor effects of cytotoxic drugs, such as apoptosis and cell cycle arrest.

Well-established evidence supports the idea that the TME may alter the chemotherapy response in favor of a drug-resistant phenotype by different mechanisms [24]. In the TME, tumor-derived exosomes mediate communication between the tumor and stromal cells, contributing to therapy evasion and tumor growth [8]. Recently, tumor-derived exosomes have been reported to play a substantial role in the differentiation of TME fibroblasts into cancer-associated fibroblasts (CAFs), which promote tumor growth, pro-angiogenic, invasive, and drug-resistant phenotypes [25,26,27]. CAFs are the most abundant stromal cell type in the TME and feature the same characteristics of myofibroblasts found during the wound healing process [28]. According to Webber et al., the transforming growth factor beta-1 (TGFβ1) transported by cancer exosomes is required to activate the tumor-promoting stroma [29]. Recent studies have identified a relationship between cancer-derived exosomal miRNAs and CAF differentiation in many types of cancer [30] (Figure 2). MiR-21 is a key regulator of oncogenic processes, which promote cell survival and the formation and activation of CAFs by regulating TGFβ1 signaling [31]. Isolated exosomes derived from patients with hepatocellular carcinoma (HCC) converted normal hepatic stellate cells to CAFs via miR-21 that downregulated the tumor suppressor gene *PTEN* and consequently upregulated the PI3K/AKT signaling pathway [32]. Similarly, exosomal miR-1247-30 from HCC cells induced CAF activation in the fibroblasts of a lung pre-metastatic niche, leading to the upregulation of pro-inflammatory genes, such as *IL1B*, *IL6*, and *IL8*, and therapy resistance to sorafenib treatment [33]. Sorafenib is a targeted therapy for advanced HCC with limited benefits for overall survival due to drug resistance, and the expression of inflammatory interleukins, such as IL-6, is directly associated with poor clinical prognosis and therapeutic inefficacy of sorafenib in HCC patients [34]. Also, a study using human melanoma exosomal miRNAs showed that miR-155 and miR-210 induced metabolic changes in human adult fibroblasts to increase aerobic glycolysis that promoted a pre-metastatic microenvironment [35]. Notably, miR-155 and miR-210 are referred to as oncogenic miRNAs that drive therapy resistance in several types of cancer [36,37,38]. 

While cancer-derived exosomal miRNAs can promote CAF differentiation, exosomal miRNAs released by CAFs in the TME play an important role in therapy resistance. Next-generation sequencing technology has shown that the exosomal transfer of miR-21 from CAFs to ovarian cancer cells inhibited apoptosis and promoted resistance to paclitaxel treatment by downregulating the expression of apoptotic peptidase activating factor (*APAF1*) [39]. Cisplatin is a platinum-based drug that exerts anticancer effects by forming adducts in the DNA of cancer cells, which activate the DNA damage response and lead cancer cells to death by apoptosis [40]. However, a recent report showed that CAF-derived exosomal miR-98-5p increased ovarian cancer cell proliferation and promoted a cisplatin-resistant phenotype by downregulating *CDKN1A*, which is a vital regulator of cell cycle arrest [41]. Interestingly, cisplatin resistance can be induced by different CAF-derived exosomal miRNAs; for example, CAF-secreted exosomal miR-196a downregulated the expression of *CDNK1B* (another key gene in the transition between the G1 and S phases of the cell cycle) in head and neck cancer cells [42]. Moreover, miR-522 derived from CAF exosomes conferred cisplatin resistance to gastric cancer cells [43]. Gemcitabine, a front-line chemotherapeutic agent for pancreatic adenocarcinoma, is known to suppress DNA synthesis in cancer cells [44]. However, exosomal miR-106b from CAFs in the pancreatic TME was reported to promote gemcitabine resistance in pancreatic cancer cells by directly downregulating *TP53INP1* expression [45]. Similarly, another study revealed that CAF-secreted exosomal miR-146a accelerated the gemcitabine-resistant phenotype in pancreatic cancer by targeting Snail pathways [46].

### 2.3. Tumor-Associated Macrophage Exosomal miRNAs Enhance Drug Resistance

Tumor-associated macrophages (TAMs) are the most abundant population of immune cells in the TME. Moreover, TAMs are extremely plastic cells that promote tumor angiogenesis, activate immunosuppression, and enhance tumor cell resistance to chemotherapy [47,48]. The size of the TAM population in the TME has been directly associated with poor prognosis in many types of cancer [49]. The chemokine C-C motif ligand 2 (CCL-2) is a chemoattractant protein for monocytes, which are secreted at high levels by cancer cells to recruit macrophages to infiltrate the tumor [50]. Recently, it was reported that colon cancer-derived exosomes carrying miR-1246 induced macrophages toward a TAM phenotype [51]. Similarly, other studies have shown that cancer-derived exosomes can carry miRNAs that promote the macrophage transition to TAMs in several types of cancers, including ovarian [52,53], bladder [54], head and neck [55], skin, and lung cancer [56]. The PI3K/AKT signaling pathway is directly associated with macrophage polarization, thereby promoting cancer migration, invasion, and drug resistance [57]. Several studies have reported that exosomes released by cancer cells modulate PI3K/AKT pathway-related genes in macrophages to promote TAM polarization [54,58,59,60].

However, in terms of drug resistance knowledge, how the exosomes released by TAMs contribute to drug resistance in tumor cells remains poorly understood. Few studies in the literature investigate the role of exosomal miRNAs derived from TAMs in drug resistance. For instance, TAMs are capable of conferring malignant phenotypes and enhancing drug resistance to epithelial ovarian cancer cells through the transfer of exosomes carrying miR-223 [61]. Another example of gemcitabine resistance was induced in pancreatic cancer cells by the delivery of miR365 through exosomes derived from TAMs [62]. Similar to CAFs, once activated, TAMs modulate the TME into an anti-inflammatory immunosuppression state by releasing exosomes carrying miRNAs in the extracellular milieu. For example, TAM-derived exosomal miR-21 leads gastric cancer cells to a cisplatin-resistant phenotype by suppressing cancer cell apoptosis and activating the PI3K/AKT signaling pathway [59], which is similar to the previously mentioned mechanism of exosomal miR-21 released by HCC cells.

### 2.4. Transfer of Drug Resistance Mediated by Cancer Stem Cells Exosomes

**Figure 3:**
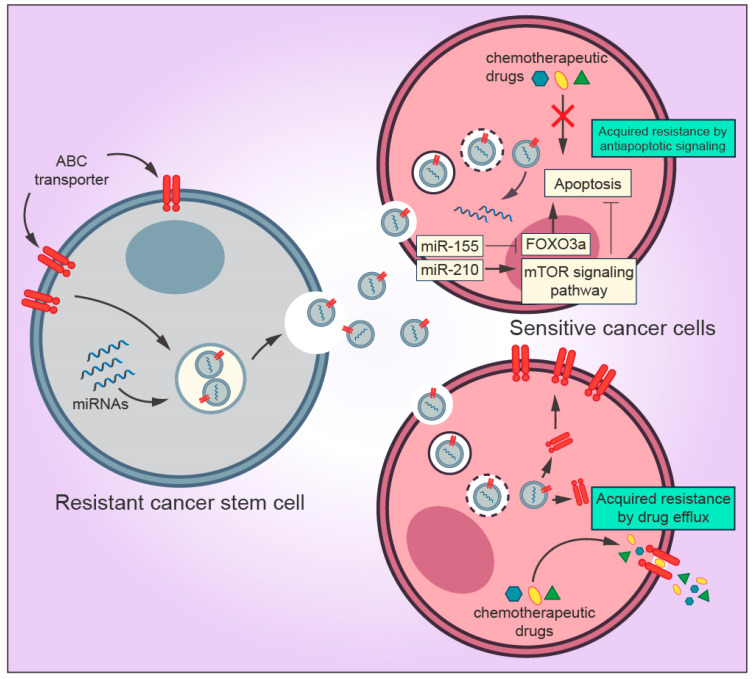
Mechanisms by which exosomes derived from cancer stem cells (CSCs) regulate drug resistance in the tumor microenvironment. Exosomal miRNAs secreted by CSCs are transferred to sensitive cancer cells and inhibit the pro-apoptotic property of FOXO3a and activates the mTOR signaling pathway, which is directly associated with inhibition of apoptosis and tumor progression, thereby blocking the drug-induced apoptosis. Exosomes can promote drug resistance to sensitive cells by transferring ABC transporters (drug efflux pumps) that actively export drugs out of the cell.

Cancer stem cells (CSCs) are the self-renewing population in the TME that exert resistance to anticancer drugs and radiotherapy [63]. CSCs can be identified through the expression of several surface markers, including high expression of CD44 (CD44^+^) and low expression of CD24 (CD24^-/low^) [64]. There is a strong connection between CSCs and tumor proliferation, metastasis, and recurrence [65]. Among many researchers, the CSC population is considered to be the source from which primary tumors develop a metastatic and resistant phenotype [66,67,68]. Recent studies have demonstrated that exosomes derived from CSCs interact with other surrounding TME and cancer cells by releasing exosomes, thereby promoting cancer progression [69,70]. Several molecular mechanisms mediated by CSCs-derived exosomes in the TME have been described, such as activation of CAF and TAM phenotype differentiation, promotion of angionesis, and induction of EMT [70,71,72] (Figure 3). However, a narrow collection of literature is available regarding the roles of CSC-derived exosomes implicated in drug resistance. Although exosomal miRNAs from cancer cells and CSCs display different profiles, they contribute to the malignant phenotype in many types of tumors [73,74,75,76]. Interestingly, few studies investigating CSC-derived exosomes have shown that miRNAs from CSC-derived exosomes are capable of transferring drug resistance to sensitive cancer cells. For instance, a study conducted by Santos and colleagues showed that miR-155 transferred by CSC-derived exosomes enhanced the resistance of breast cancer cells to doxorubicin and paclitaxel treatment and induced the acquisition of EMT phenotype [77]. More recently, using in vitro and in vivo approaches, Yang et al. showed that gemcitabine-resistant pancreatic CSCs exosomes containing high levels of miR-210 horizontally transferred the resistant phenotype to gemcitabine-sensitive pancreatic cancer cells, thereby inhibiting apoptosis and promoting proliferation by targeting the mTOR signaling pathway [37].

The most prominent property of CSCs is their ability to resist conventional drug treatment regimens as mentioned earlier. Despite the horizontal transfer of miRNAs to neighboring cells mediated by exosomes, CSCs can also enhance multidrug resistance in many types of tumors due to the overexpression of ATP-binding cassette (ABC) transporters [70,78]. Members of the ABC protein family, such as ABCB1 (also known as P-glycoprotein), ABCC1, and ABCG2, function as efflux transporters of a wide range of molecules, including anticancer drugs, to the extracellular milieu [79]. Several cytotoxic drugs, such as cisplatin, paclitaxel, and doxorubicin, are substrates to ABC transporters [80]. ABC transporters reduce the intracellular concentrations of chemotherapeutic drugs to suboptimal levels in CSCs, consequently leading the cells to survive under treatment conditions [78].

Recently, it was found that ABC drug efflux pumps are incorporated in the cargo of resistant cell exosomes and transferred to drug-sensitive cells [81]. Resistant cell exosomes transporting P-glycoprotein to neighboring cells is the most studied multidrug resistance mechanism mediated by ABC transporters in several types of cancer [8]. Docetaxel-resistant MCF-7 breast cancer cells promoted drug resistance by the exosomal delivery of P-glycoprotein to docetaxel-sensitive MCF-7 cells [82]. Likewise, using in vitro and preliminary clinical studies, Corcoran et al. observed that P-glycoprotein released by prostate cancer exosomes induced phenotypic changes toward cell proliferation and conferred a significant increase in docetaxel resistance to sensitive cells [83]. The drug resistance acquisition was also observed in ovarian cancer, in which A2780 human ovarian cancer cells became unresponsive to paclitaxel after the exosomal transfer of P-glycoprotein from paclitaxel-resistant A2780 cells [84]. Moreover, a recent study identified six different miRNAs associated with drug resistance (miR-204-5p, miR-139-5p, miR-29c-5p, miR-551b-3p, miR-29b-2-5p, and miR-204-3p) delivered by doxorubicin-resistant cancer exosomes. The authors reported that doxorubicin-resistant lung cancer and chronic myeloid leukemia cells transferred exosomal miRNAs responsible for P-glycoprotein regulation, thereby enhancing drug resistance in their sensitive counterparts [85]. 

### 2.5. EMT Mediated by Exosomal miRNAs

EMT is the process by which carcinoma cells lose their apical-basal polarity and cell junctions to acquire mesenchymal features, such as elongated morphology, resulting in cancer cells with high plasticity and increased motility [86,87]. Due to the highly invasive acquired phenotype, EMT cells are directly associated with cancer progression, metastasis, and drug resistance in different types of cancer [88]. Stromal cells, such as CAFs, play an important role in the TME to support EMT-driven drug resistance [89]. CAFs can regulate the EMT process via the paracrine release of pro-inflammatory cytokines, thereby promoting acquired drug resistance in cancer cells undergoing EMT [90,91]. Additionally, EMT has a close connection to CSCs, including expression of the same surface markers CD44^+^ and CD24^-/low^ [92] as well as activation of similar signaling pathways, such as Hedgehog, Wnt, and Notch [88]. As discussed previously, CSCs promote drug resistance due to excessive drug efflux mediated by ABC transporters; similarly, cells undergoing EMT have been reported to express high levels of ABC transporters, including P-glycoprotein [93,94].

In recent years, accumulating evidence has shown that cancer-derived exosomes can modulate the EMT process in the TME [16,95,96]. Xiao and colleagues provided strong evidence of cancer exosomes promoting EMT. In their study, melanoma-derived exosomes induced EMT in primary melanocytes by transferring miRNAs directly associated with EMT regulation, such as let-7a and miR-191 [97]. Exosomes released from drug-resistant endothelial cells triggered the EMT process and promoted doxorubicin resistance in nasopharyngeal cancer cells [98]. Emerging studies are revealing the influence of exosomal miRNAs in primary tumor cells by activating or stabilizing EMT. Recently, a multidrug-resistant HCC cell line (resistant to sorafenib, gemcitabine, oxaliplatin, and 5-fluorouracil) delivered exosomal miR32-5p to HCC-sensitive cells, leading to activation of the PI3K/AKT signaling pathway and inducing multidrug resistance via angiogenesis and EMT [99]. Exosomal miR-155-5p released by paclitaxel-resistant gastric cancer MGC-803 cells induced the EMT malignant phenotype and enhanced paclitaxel resistance via the suppression of *GATA3* and *TP53INP1* in parental sensitive cells [100]. In another study, normal intestinal cells were transfected with miR-128-3p, and their exosomes were co-cultured with oxaliplatin-resistant colorectal cancer cells. The results revealed that miR-128-3p suppressed EMT by upregulating E-cadherin levels and increasing the intracellular oxaliplatin concentration in colorectal cancer cells [101]. These findings provide a novel exosome-based treatment strategy.

### 2.6. Autophagy Induced by Cancer Exosomal miRNAs

Autophagy is a catabolic process responsible for the elimination of damaged or excessive macromolecules or organelles to maintain homeostasis and metabolic sufficiency [102]. Autophagy also plays a dual role in cancer by promoting tumor growth and increasing tumor resistance to therapy [103]. During cancer initiation, autophagy is frequently upregulated, thereby conferring a high energy supply and cell survival [104]. A high level of autophagy is directly associated with a hypoxic TME, which leads to oxidative stress, delays apoptotic cell death, and contributes to therapy resistance, a phenomenon called cytoprotective autophagy [105,106]. The crosstalk between cancer exosomes and autophagy has attracted substantial attention. According to some authors, this interaction is dynamic and directly associated with the tumor’s needs [107,108]. For example, under cellular stress, exosomes and autophagy can be upregulated in cancer cells to confer an adaptative response. On the other hand, cancer-derived exosomes can also promote the formation of reactive oxygen species (ROS) and upregulate autophagy in recipient cells to promote the secretion of tumor growth factors [109]. Finally, under a drug treatment regimen, cancer cells release exosomes that upregulate cytoprotective autophagy in recipient cells, leading to a resistant phenotype [110]. 

Many other studies unrelated to cancer have shown that exosomal miRNAs can control autophagy in recipient cells by targeting specific autophagy-related signaling pathways [111,112]. Currently, there are a handful of studies investigating the role of exosomal miRNAs as autophagy mediators in therapy resistance. For instance, exosomal miR-425-3p derived from cisplatin-resistant non-small cell lung cancer (NSCLC) cells decreased responsiveness to cisplatin via targeting the AKT1/mTOR signaling pathway, consequently leading to upregulation of autophagic activity [113]. Similar results were observed in a study by Ma et al., in which cisplatin-resistant NSCLC exosomal miR-425-3p facilitated autophagic activation and conferred cisplatin resistance in sensitive cells by also targeting the AKT1/mTOR signaling pathway [114]. Given these results, we can assume that miR-425-3p is a promising biomarker candidate for predicting the cisplatin response in NSCLC. More recently, cells from trastuzumab-resistant breast cancer patients showed a significantly lower expression of exosomal miR-567 in comparison with sensitive patients. Using in vitro and in vivo approaches, it was found that miR567 was downregulated in trastuzumab-resistant cells compared to sensitive cells. Moreover, miR-567 inhibited trastuzumab-induced autophagy and reverted the acquired drug resistance [115]. These results indicate that cancer cells can downregulate exosomal miRNAs that control anticancer mechanisms and drug sensitivity in neighboring cells.

## 3. Enhanced Drug Efficacy Mediated by Inhibitors of Exosome Biogenesis

As discussed above, exosomal miRNAs can modulate several different molecular mechanisms and signaling pathways of neighboring cancer cells or TME-associated cells. This influence can either induce cancer growth, metastasis, and therapy resistance or inhibit the expression of tumor suppressors [116]. Tumor cells release more exosomes into the TME than non-tumoral cells, which means that the circulating level of exosomes in the TME is abundant [117,118]. According to Sharma, a reasonable strategy to overcome the drug resistance mediated by exosomes and exosomal miRNAs involves blocking exosome biogenesis [18]. Although exosome biogenesis is not completely understood [119], a few compounds have been identified as potential inhibitors of exosome generation. GW4869 is a pharmacological agent that showed promising results as an inhibitor of exosome biogenesis in different types of cancer, including prostate [120], colorectal [121], and pancreatic cancer [46]. Moreover, the inhibition of exosome biogenesis induced by GW4869 in combination with bortezomib showed a strong anti-tumor response in vivo [122]. For instance, an interesting study reported several other compounds as promising mediators of exosome biogenesis and secretion in prostate cancer cells, including farnesyl transferase inhibitors, such as tipifarnib and manumycin A, and azole antifungals, such as climbazole, ketoconazole, and neticonazole [123]. Also, a recent study showed that manumycin A is capable of inhibiting exosome biogenesis and secretion by targeting the inhibition of the Ras/Raf/ERK signaling pathway in prostate cancer cells [124]. Sulfisoxazole, an antibiotic drug, was recently identified as an inhibitor of exosome biogenesis in breast cancer cells. Additionally, sulfisoxazole in combination with docetaxel reduced the proliferation and metastasis in mouse models of cancer cells xenograft [125]. Moreover, a combination treatment of chloramidine with bisindolylmaleimide was reported to inhibit exosome biogenesis in resistant prostate and breast cancer cells. The synergistic effect of these compounds targeted specific extracellular vesicle biogenesis pathways and enhanced the efficacy of 5-fluorouracil, which leads cancer cells to apoptosis [126]. Likewise, indomethacin, a non-steroidal anti-inflammatory drug, was revealed to be an effective inhibitor of ABC transporter expression, consequently increasing the anticancer effects of pixantrone and doxorubicin in B-cell lymphoma cells in vitro [127]. As stated earlier, the transport of ABC transporters by exosomes is crucial to maintain the drug-resistant phenotype. Taken together, these studies highlight the inhibition of exosome biogenesis as a promising approach to improve drug efficacy and overcome therapy resistance as well as underscore the need for more investigation concerning the aspects of exosome biogenesis and exosome-mediated drug resistance.

## 4. Conclusions

Tumor heterogeneity comes from different interactions as well as intra- and extracellular alterations, resulting in a dynamic and reactive environment that promotes tumor growth and suppresses therapeutic approaches. Here, we presented a plethora of evidence that exosomes are key extracellular mediators of the communication between tumor cells and the TME during different cancer stages, including cancer progression and metastasis. Cancer-derived exosomes carry miRNAs that are crucial to promoting a switch in tumor heterogeneity to a resistant phenotype (Table 1). Moreover, cancer stem cells and non-cancer cells in the TME contribute to drug resistance by releasing exosomal miRNAs that have different effects on the target cells. Also, exosomal miRNAs can induce resistance to cytotoxic drugs as well as molecular target-specific drugs. For example, miR-155 has been reported to promote resistance to doxorubicin and paclitaxel treatment in multiple kinds of cancer. On the other hand, several distinct miRNAs induced gemcitabine resistance in pancreatic cancer cells. Furthermore, some miRNAs seem to regulate therapy resistance in a tumor-specific manner; for example, different studies have demonstrated that miR-128-3p and miR-425-3p act as drug resistance mediators in colorectal and lung cancer, respectively. For this reason, exosomal miRNAs can be used as potential cancer biomarkers for diagnosis and prognosis in a broad or tumor-specific way. Novel approaches to challenging the drug resistance barrier created by exosomes are emerging, such as the use of exosome biogenesis inhibitors in synergy with other therapeutic drugs. In summary, we conclude that the exosome investigation has emerged as a promising field in cancer research and provides tremendous insights into many aspects of cancer communication and acquired drug resistance. However, important questions remain, and further studies are needed to better understand the role and targets of miRNAs released by exosomes in acquired drug resistance. Future research on exosomal miRNAs and the TME in drug resistance will not only identify new exosomal miRNAs and their crucial functions during tumor progression but also pinpoint targets for the prevention, diagnosis, and development of new therapeutic strategies. 

## Figures and Tables

**Table 1:** Function and effects of exosomal miRNAs involved in tumor drug resistance.

Exosome Origin	Exosomal miRNA	Function(s)/Effect(s)	Ref.
Breast cancer cells	miR-1246 miR-423-5p	Involved in increased cisplatin, docetaxel, epirubicin, and gemcitabine resistance in sensitive breast cancer cells	[128,129]
Colorectal cancer cells	miR-128-3p miR-31-5p	Involved in transferred oxaliplatin resistant phenotype to sensitive colorectal cancer cells	[101,130]
Gastric cancer cells	miR-106a-5p, miR-421	Involved in promoted 5-fluorouracil resistance	[131]
Glioblastoma cells	miR-1238 miR-151a	Involved in transferred temozolomide resistance to sensitive glioblastoma cells	[132,133]
Lung cancer cells	miR-100-5p	Involved in transferred the cisplatin-resistant phenotype to sensitive lung cancer cells	[134]
Lung cancer cells	miR-204-5p, miR-139-5p, miR-29c-5p, miR-551b-3p, miR-29b-2-5p, and miR-204-3p	Transferred doxorubicin-resistant phenotype to sensitive lung cancer cells	[85]
Non-small cell lung cancer	miR-425-3p, miR-214	Induced cisplatin and gefitinib resistance in neighboring cells, respectively	[113,135]
Ovarian cancer cells	miR-1246	Involved in conferred paclitaxel resistance to neighboring cancer cells	[136]
Pancreatic cancer cells	miR-155, miR-210	Involved in promoted ROS detoxification and gemcitabine resistance	[37,137]
CAFs ^1^	miR-21	Involved in paclitaxel resistance transfer to ovarian cancer cells	[39]
CAFs	miR-106b, miR-146a	Involved in increased pancreatic cancer cell proliferation and gemcitabine chemoresistance	[45,46]
CAFs	miR-16, miR-148a	Involved in promoted breast cancer cell migration and metastasis	[138]
CAFs	miR-196a	Involved in conferred cisplatin resistance in head and neck cancer cells	[42]
TAMs ^2^	miR-365	Involved in enhanced gemcitabine resistance in pancreatic cancer cells	[62]
TAMs	miR-21	Involved in suppressed cell apoptosis and enhanced cisplatin resistance in gastric cancer cells	[59]
TAMs	miR-223	Involved in induced cisplatin-resistant phenotype in ovarian cancer cells	[61]

^1^ Cancer-associated fibroblast. ^2^ Tumor-associated macrophages
